# Polyphenolic protection: the role of mangiferin in mitigating neurodegeneration and neuroinflammation

**DOI:** 10.1007/s10787-025-01854-3

**Published:** 2025-07-22

**Authors:** Aya M. Mustafa, Ghadir A. Bastawesy, Shymaa Hatem, Roxane Abdel-Gawad Moussa, Dina M. Hal, Mariam H. Fawzy, Mahmoud A. Mansour, Mohamed S. Abd El Hafeez

**Affiliations:** 1 Department of Pharmacology and Toxicology, Faculty of Pharmacy, Egyptian Russian University, Badr City, Cairo, 11829 Egypt; 2 Biochemistry Department, Faculty of Pharmacy, Egyptian Russian University in Cairo, Badr City, Cairo, 11829 Egypt; 3 Department of Pharmaceutics and Pharmaceutical Technology, Faculty of Pharmacy, Future University in Egypt, Cairo, Egypt; 4 Department of Pharmaceutics and Industrial Pharmacy, Faculty of Pharmacy, Ain Shams University, Cairo, Egypt; 5 Department of Pharmacognosy, Faculty of Pharmacy, Suez Canal University, Ismailia, 41522 Egypt; 6 Department of Pharmacognosy, Faculty of Pharmacy, Egyptian Russian University, Cairo-Suez Road, Badr City, Cairo, 11829 Egypt; 7 Department of Pharmacognosy, College of Pharmacy, University of Kut, Al Kut, Wasit, 52001 Iraq

**Keywords:** Mangiferin, Synthesis, Neurological disorders, Drug delivery systems, Bioavailability, Pharmacokinetics

## Abstract

Mangiferin, a polyphenol of natural occurrence predominantly present in *Mangifera indica*, has attracted significant interest because of its multiplicity of pharmacological activities, of which its neuroprotective activity is of particular interest. Herein, the review delves into the biosynthetic mechanisms, structural properties, and action mechanisms responsible for the therapeutic value of mangiferin in neurological disorders. Of special note is that mangiferin has antioxidative, anti-inflammatory, and anti-apoptotic activities, which are responsible for its effectiveness in reducing cognitive impairments and neuronal damage in disorders such as Alzheimer’s disease, Parkinson’s disease, Huntington’s disease, stroke, epilepsy, depression, anxiety, and general cognitive decline. Though being of low bioavailability, more recent approaches such as chemical derivatization, nanoparticle-mediated drug delivery, and intranasal delivery have the promise to enhance its central nervous system (CNS) delivery. By combining preclinical and mechanistic studies, the review herein highlights the potential of mangiferin as a multitherapeutic neuroprotectant and addresses new approaches to facilitate its clinical application.

## Introduction

Neurodegenerative diseases and psychiatric disorders are the chief causes of worldwide health morbidity due to their complex pathogenesis and limited curative therapies (Gadhave et al. 2024). Oxidative stress, neuroinflammation, and mitochondrial dysfunctions form the core of their etiology. Due to this fact, multitarget neuroprotective natural compounds have become more prominent, Mangiferin (Abbas et al. 2025), which is a glucosylxanthone with most of its origins in *Mangifera indica*, has exhibited considerable pharmacological activity in an extensive range of biological platforms (Zivković et al. 2024).

With an xanthone skeleton possessing a number of hydroxyl groups and an attached glucose unit, mangiferin possesses numerous effects like antioxidant, anti-inflammatory, antidiabetic, anticancer, and neuroprotective activities (Zivković et al. 2024). This has been proven by huge experimental evidence with in vitro and in vivo models of Alzheimer’s, Parkinson’s, Huntington’s disease, stroke, epilepsy, and mood disorders. Its capacity to regulate multiple key molecular pathways such as NF-κB, NLRP3 inflammasome, PI3K/Akt, and Nrf2/ARE further establishes its therapeutic value (Zhang et al. 2024b).

Nonetheless, clinical translation of mangiferin is obfuscated by its poor bioavailability with limited solubility and complete metabolism (Zivković et al. 2024). Thus, there is a need for the design of new delivery systems and chemical analogs to maximize its complete neurotherapeutic potential. In this review, it is hoped that current evidence of the neuroprotective effects of mangiferin will be synthesized and the avenues for future research mapped out in CNS disorders (Ehianeta et al. 2016).

## Biosynthesis of mangiferin

Mangiferin is a glucosylxanthone. Biosynthesis of mangiferin occurs by the synthesis of xanthone aglycone and glycosylation of xanthone core with a glucose moiety. xanthones are present in lower and higher plants as well as fungi and lichens. The biogenesis of these compounds varies according to the oxygenation pattern. This biosynthesis was derived from a combination of the shikimic acid pathway and acetate-malonate polyketide pathway (Remali et al. 2022). In more detail, it was assumed that the acetate-malonate polyketide pathway produced ring A, while the shikimic acid pathway supplied ring B (Peres et al. 2000; Dutta et al. 2023). First, the phenylalanine ammonia-lyase (PAL) catalyzes the conversion of phenylalanine to cinnamic acid. The cinnamic acid further generates p-coumaric acid then caffeic acid. The 1,3,6,7-tetrahydroxyxanthone, norathyriol, is more likely to be produced when maclurin directs phenolic oxidative coupling between the OH at the C2 position of the benzophenone ring A and the C6` of the benzophenone ring B. This condensation between shikimate and acetate-derived moieties forms the benzophenone intermediate (Ehianeta et al. 2016).

**Fig. 1:**
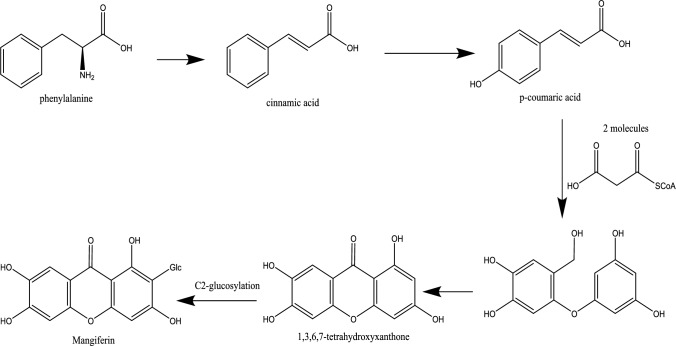
Biosynthesis pathway of mangiferin

This biogenetic route of mangiferin was confirmed by a retrobiosynthetic approach using the aerial parts of *Anemarrhena asphodeloides*. This method based on feeding ^14^C-labeled shikimate and malonate derivatives (López-Cárdenas et al. 2023). According to the data, maclurin is the biogenetic precursor of norathyriol, which is produced by the condensation of a C6-C3 shikimate precursor with two molecules of malonate in a convergent manner (Fujita and Inoue 1980a). Three observations further bolstered this proposal: (a) commercial maclurin contains norathyriol as an impurity; (b) maclurin undergoes chemical or photochemical oxidation to produce norathyriol in vitro; and (c) maclurin and norathyriol have previously been isolated from *Symphonia globulifera* heartwood (Fujita and Inoue 1981). Moreover, this proposal confirmed by another evidence using a method based on the oxidation of maclurin into norathyriol by direct yeast catalyst. The result strongly suggests that direct phenolic oxidative coupling of maclurin produced norathyriol. The glycosylation of xanthone was investigated to predict the introduction of a glucose unit was occurred at which stage of the biosynthetic pathway maclurin or norathyriol (Fujita and Inoue 1980b). According to experiments, C-glucosylation took place at the maclurin stage before norathyriol was formed (Tanaka et al. 1984). Consequently, these results showed that 3-C-glucosylmaclurin is a possible intermediate in the biosynthesis of mangiferin as well as other related *C*-glucosylxanthones. Indeed, phenolic oxidative ring closure of 3-*C*-glucosylmaclurin can provide four isomeric *C*-glucosylxanthones: mangiferin, isomangiferin, 1,3,5,6-tetrahydroxy-2-C-glucoside and 1,3,5,6-tetrahydroxy-4-C-glucoside. This biosynthetic route can explain the cooccurrence of mangiferin and isomangiferin with 1,3,5,6-tetrahydroxyxanthone-C-glucosides as well as the formation of homomangiferin by 3-O-methylation of mangiferin in a later stage (Ghosal and Chaudhuri 1973) (Fig. 1).

### Structure-activity relationship of mangiferin

The structure–activity relationship (SAR) of mangiferin has been extensively investigated owing to its diverse pharmacological activities, which include antioxidant, anti-inflammatory, antidiabetic, anticancer, and neuroprotective effects (Imran et al. 2017). These activities are associated with its structure, as the backbone of mangiferin is a xanthone, characterised by a tricyclic aromatic system with a dibenzo-γ-pyrone configuration. Additionally, a C-glucosyl linkage is present at position 2 of the xanthone ring, accompanied by four hydroxyl groups on the aromatic rings at positions 1, 3, 6, and 7 (Benard and Chi 2015). The quantity and arrangement of hydroxyl groups are essential for its antioxidant and radical scavenging efficacy. Particularly, hydroxyl groups at positions 1 and 3 play a crucial role in metal chelation and the neutralisation of reactive oxygen species (ROS) (Kaurav et al. 2023). C-Glucosylation at C2 enhances stability against hydrolysis. β-D-glucose improves aqueous solubility and bioavailability. The elimination or modification of the glucose unit frequently diminishes anti-inflammatory and antiviral efficacy (Ain et al. 2023). The planar aromatic xanthone core is crucial for DNA intercalation, which is pertinent to anticancer efficacy. Derivatives in which this core is altered or disrupted forfeit their significant biological features (Benard and Chi 2015). The methylation or acylation of hydroxyl groups can modify lipophilicity, possibly enhancing cellular permeability, but frequently diminishes activity. The antioxidant activity diminished when the hydroxyl groups in the glucose or norathyriol moieties were subjected to methylation or acetylation. The antioxidant efficacy of norathyriol is highly contingent upon its hydroxyl groups. The antioxidant capabilities are decreased when –OH groups are methylated or acetylated at the C3, C6, and C7 positions. Only R7 requires modification to –COCH = CH-C_6_H_5_ to substantially diminish the antioxidant capabilities (Dar et al. 2005). The data indicate that the –OH groups are essential for antioxidant activities, suggesting that metal chelation significantly contributes to the actions of mangiferin. The ability to form stable mangiferin-Fe2 + /Fe3 + complexes prevents lipid peroxidation and the Fenton reaction. Mangiferin exhibited antidiabetic properties via many pathways. These pathways encompass the enhancement of *β*-cell proliferation, pancreatic islet regeneration, and the healing of severe islet damage induced by STZ. The esterification of the –OH groups of MGF with –COCH₃, –COCH₂CH₃, and –COCH₂CH₂CH₃ enhanced the pharmacophore’s capacity to restore injured islets and doubled the hypoglycemic effects (Klaman et al. 2000). Another mechanism involves the suppression of protein-tyrosine phosphatase 1B (PTP1B), which significantly promotes the dephosphorylation of the active insulin receptor, hence diminishing insulin signalling (Elchebly et al. 1999). Replacing hydroxyl groups at the C3, C6, and C7 positions with (CH_2_)_9_ CH3 results in a derivative that demonstrated 100% inhibition of PTP1B at 50 μM (Xue-Jian et al. 2013). This signifies a substantial improvement, given that the parent MGF inhibited just 24.07% of PTP1B activity at 500 μM (Hu et al. 2007). The dimerization of mangiferin enhanced its antiviral activity relative to the parent mangiferin (Abdel-Mageed et al. 2014). The antipyretic efficacy of mangiferin was marginally enhanced by exchanging 5-H with 5-CH₂R (R = diphenylamine) (Singh et al. 2011).

## Distribution of mangiferin

Mangiferin (Table 1) is mainly derived from *Mangifera indica*, but it can also be obtained from a variety of other plants (Jyotshna et al. 2016).

**Table 1:** Distribution of mangiferin

Plant name	Family	Part used	Amount	Method used	Refs
*Mangifera indica* (XH-2)	Anacardiaceae	Seed Kernel	1.04 mg/g	HPLC	Luo et al., (2012)
*Mangifera indica* (XH-2)	Anacardiaceae	Peel	0.67 mg/g	HPLC	Luo et al., (2012)
*Mangifera indica* (ZHM)	Anacardiaceae	Peel Seed Kernel	7.34 mg/g, 2.43 mg/g	HPLC, HPLC	Luo et al., (2012)
*Mangifera indica* (LPM)	Anacardiaceae	Peel Seed Kernel	7.49 mg/g, 0.67 mg/g	HPLC, HPLC	Luo et al., (2012)
*Mangifera indica*	Anacardiaceae	(leaves) The Thawai cultivar leaves. The Namdokmai,	1.94 ± 0.13–13.79 ± 0.84% dry wt	ELISA	Yusakul et al., (2012)
*Mangifera indica* Dashahri	Anacardiaceae	Mangiferin content in hot methanolic extract from dry leaves	13.48%	HPLC	Saxena et al., (2024)
*Mangifera indica* Dashahri	Anacardiaceae	Mangiferin content in dry leaves	2.34%	HPLC	Saxena et al., (2024)
*Mangifera indica* Chausa	Anacardiaceae	Mangiferin content in hot methanolic extract from dry leaves	13.22%	HPLC	Saxena et al., (2024)
*Mangifera indica* Chausa	Anacardiaceae	Mangiferin content in dry leaves	3.00%	HPLC	Saxena et al., (2024)
*Mangifera indica* Langra	Anacardiaceae	Mangiferin content in hot methanolic extract from dry leaves	9.20%	HPLC	Saxena et al., (2024)
*Mangifera indica* Langra	Anacardiaceae	Mangiferin content in dry leaves	0.96%	HPLC	Saxena et al., (2024)
*Mangifera indica* Lucknow safeda	Anacardiaceae	Mangiferin content in hot methanolic extract from dry leaves	11.48%	HPLC	Saxena et al., (2024)
*Mangifera indica* Lucknow safeda	Anacardiaceae	Mangiferin content in dry leaves	1.36%	HPLC	Saxena et al., (2024)
*Mangifera indica* Gola	Anacardiaceae	Mangiferin content in hot methanolic extract from dry leaves	12.70%	HPLC	Saxena et al., (2024)
*Mangifera indica* Gola	Anacardiaceae	Mangiferin content in dry leaves	1.46%	HPLC	Saxena et al., (2024)
*Cyclopia intermedia*	Fabaceae	Aerial parts	1.69 g per 100 g	RP-HPLC	Joubert et al., (2003)
*Cyclopia maculata*	Fabaceae	Aerial parts	1.63 g per 100 g	RP-HPLC	Joubert et al., (2003)
*Cyclopia sessiliflora*	Fabaceae	Aerial parts	1.04 g per 100 g	RP-HPLC	Joubert et al., (2003)
*Cyclopia genistoides*	Fabaceae	Aerial parts	3.61 g per 100 g	RP-HPLC	Joubert et al., (2003)
*Swertia franchetiana*	Gentianaceae	Whole plant	2.00–6.00 mg/g	HPLC	Kshirsagar et al., (2016)
*Swertia mussotti*	Gentianaceae	Whole plant	15.10–44.21 mg/g	HPLC	Kshirsagar et al., (2016)
*Swertia punicea*	Gentianaceae	Whole plant	0.42–8.86 mg/g	HPLC	Kshirsagar et al., (2016)
*Swertia chirayita*	Gentianaceae	Whole plant	0.69–3.03 mg/g	HPLC	Kshirsagar et al., (2016)
*Swertia densifolia*	Gentianaceae	Whole plant	6.02 mg/g	HPLC	Kshirsagar et al., (2016)
*Canscora perfoliata*	Gentianaceae	Whole plant	1.407 ± 0.1%	HPTLC	Deepak et al., (2019)
*Aphloia theiformis*	Aphloiaceae	leaves	Not specified	HPLC UPLC–QTOF–MS	Grauzdytė et al., (2020)

Mangiferin is derived primarily from *Mangifera indica* but is also found in other plants. In *Mangifera indica* (XH-2), seed kernel contains 1.04 mg/g and peel contains 0.67 mg/g of mangiferin by HPLC quantitation. In the ZHM cultivar of *M. indica*, 7.34 mg/g is in the peel and 2.43 mg/g in the seed kernel. The LPM cultivar contains 7.49 mg/g in the peel and 0.67 mg/g in the seed kernel, also determined by HPLC (Luo et al. 2012).

Leaves of different cultivars of *M. indica* have leaves with varying content of mangiferin. The Thawai cultivar, which is rare, has the highest content of 13.79 ± 0.84% dry weight, whereas the Thai cultivar Namdokmai, which is more common, has 12.41 ± 0.60% dry weight. The mangiferin content in leaves of all the cultivars studied ranged from 1.94 ± 0.13 to 13.79 ± 0.84% when measured by ELISA (Yusakul et al. 2012).

Other cultivars such as Dashahri, Chausa, Langra, Lucknow Safeda, and Gola have been quantitated for mangiferin content in hot methanolic extracts and dry leaf state by HPLC. Dashahri yielded 13.48% mangiferin in hot methanolic extracts and 2.34% in dry leaves. Chausa yielded 13.22% in methanolic extract and 3.00% in dry leaves. Langra yielded 9.20% in methanolic extract and 0.96% in dry leaves. Lucknow Safeda yielded 11.48% in extract and 1.36% in dry leaves. Gola exhibited 12.70% in extract and 1.46% in dry leaves (Saxena et al. 2024).

In the genus Cyclopia of the family Fabaceae, aerial parts of *C. intermedia, C. maculata, C. sessiliflora,* and *C. genistoides* contain mangiferin content ranging from 1.04 to 3.61 g per 100 g as analyzed by RP-HPLC. Specifically, *C. genistoides* had the most at 3.61 g/100 g (Joubert et al. 2003).

Within the Gentianaceae family, whole plants of *Swertia franchetiana, Swertia mussotti, Swertia punicea, Swertia chirayita,* and *Swertia densifolia* were analyzed by HPLC. Their mangiferin content was quite different: *S. franchetiana* (2.00–6.00 mg/g), *S. mussotti* (15.10–44.21 mg/g), *S. punicea* (0.42–8.86 mg/g), S. chirayita (0.69–3.03 mg/g), and S. densifolia (6.02 mg/g) (Kshirsagar et al. 2016).

The whole plant of *Canscora perfoliata* (Gentianaceae) contained 1.407 ± 0.1% mangiferin content as approximated by HPTLC. Mangiferin was also found in leaves of *Aphloia theiformis* (Aphloiaceae), but the quantity was not specified. It was quantified using HPLC and UPLC–QTOF–MS (Kshirsagar et al. 2016).

## Effect of mangiferin on neurodegenerative diseases

**Fig. 2:**
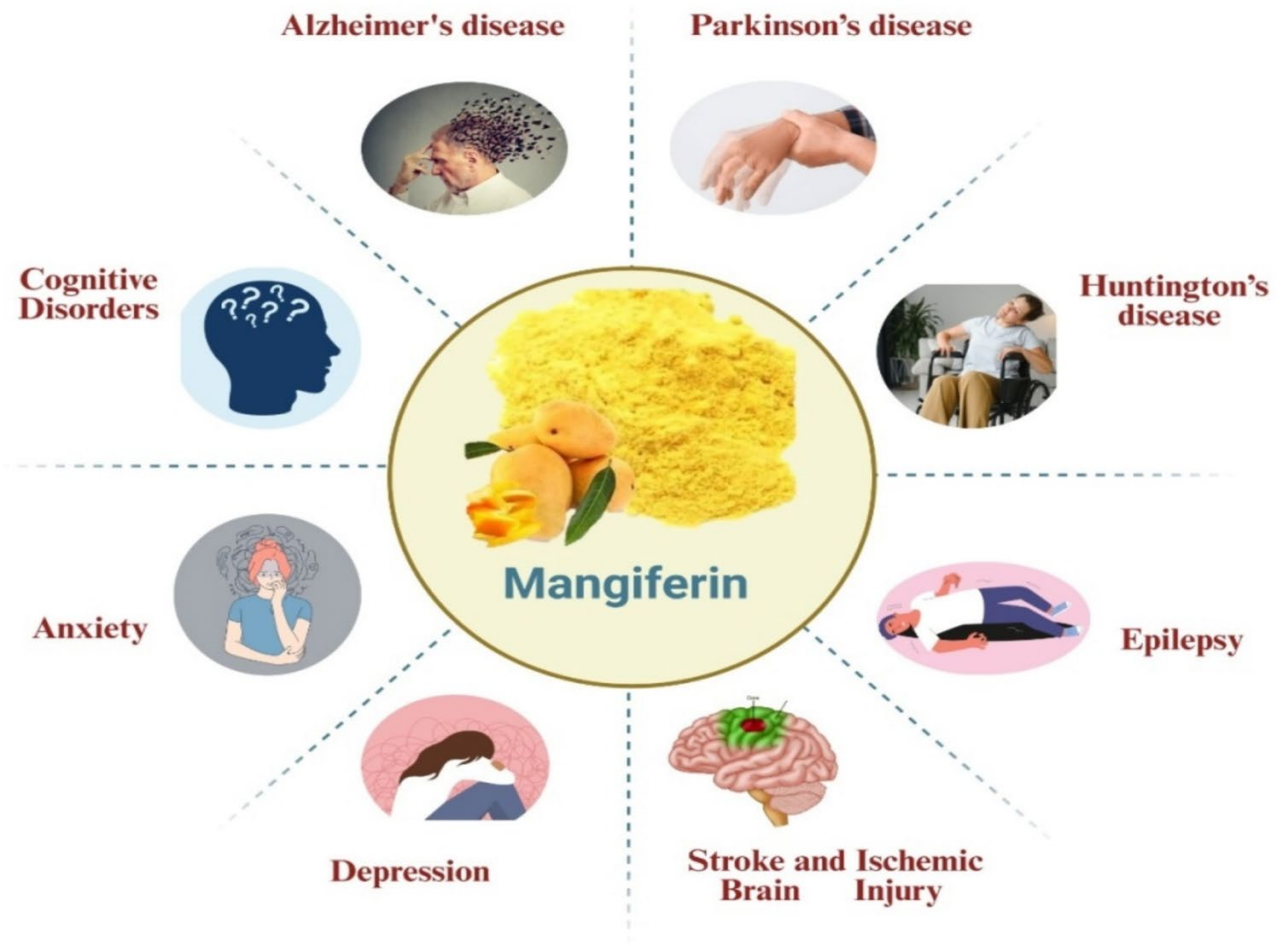
Pharmacological effect of mangiferin

Mangiferin is considered an effective molecule for different neuro diseases as shown in Fig. 2 and Table 2.

**Table 2:** Effect of mangiferin on neurological disorder

Neurological Disorder	Experimental study In vitro using In vivo on cell lines animal model		_Dose_ In vitro In vivo		Mechanism	References
Alzheimer’s disease	Murine BV2 microglial cells		50–150 μg/mL		↓ NF-κB and NLRP3 signaling pathway	Lei et al., (2021)
		Male Swiss albino mice		(20 and 40 mg/kg, p.o) daily for 21 days	↓cognitive deficits, ↓hippocampal BDNF depletion	Kasbe et al., (2015)
		Swiss Albino mice		(40, 20 and 10 mg/kg, i.p.) for 14 days	Improve the learning and memory	Hao et al., (2019)
		Male SAMP8 mice		(100 and 200 mg/kg, p.o) daily for 60 days	• Improve learning and memory • keep hippocampus cell integrity • ↓reduce brain Aβ accumulation • ↓brain LPO level	Du et al., (2019)
		Male Albino mice		(10, 20, 40 mg/kg, p.o)	• Improve memory deficits • ↓AChE • ↓NF-kB	Jung et al., (2009)
	Neuro-2A	Male sprague–dawley rats	(10 and 20 μg/ml)	50 and 100 mg/kg p.o	• ↓AChE • ↓5-LOX	Sethiya and Mishra, (2014)
		APPswe/PS1d9 (APP/PS1) mice		50 mg/kg/day Orally for 22 weeks	• Improve central pathology • ↓Cognitive deficits	Infante-Garcia et al., (2017)
Parkinson’s disease	SH-SY5Y cells				• ↓oxidative damage • ↓neuro-inflammation	Zhou et al., (2023)
		Male C57BL/6 mice		(10, 20, 40 mg/kg, p.o) for 14 days	• ↓oxidative stress • ↓apoptosis	Kavitha et al., (2013)
	PC12 cell line	Male C57BL/6 mice	(1 μM, 10 μM, 20 μM, 50 μM, 100 μM)	30 mg/kg/day for 14 days intraperitoneal	• ↓α-synuclein • ↓AKR1C3 • ↑ Wnt/β-catenin	Huang et al., (2024)
		Male Wistar rats		(15 µg, 30 µg, and 45 µg) for 28 days through the cannula	• ↑ Locomotor activity • ↓oxidative stress • ↓inflammatory markers	Tiwari et al., (2021)
		Wild-type AB zebrafish		25, 50, 100 mg/L	• ↓mitochondrial-related oxidative stress	Qin et al., (2024)
		Male Wistar rats		45 μg for 28 days	• Anti-inflammatory • Anti-parkinsonism	Tiwari et al., (2022)
		Male Wistar rats		45 μg for 28 days	• Anti-inflammatory • Anti-oxidant	Tiwari et al., (2024a)
		C57BL/6 mice		20 mg/kg	• Recovering mitochondrial ultrastructure • Enhancing ATP contents	Wang et al., (2022)
	N2A cells		100 µM		• ↓oxidative stress	Amazzal et al., (2007)
	SK-N-SH neuroblastoma cell line		(2.5, 5, 10, 20 and 40 μg/ml)		• ↑mitochondrial membrane potential • ↓ apoptosis	Kavitha et al., (2014)
	PC12 cells		20 and 80 μM		• ↓oxidative damage	Shi et al., (2017)
Huntington’s disease		Mixed-gender Wistar rats		(10, 20 mg/kg, p.o.) for 14 days	• ↓oxidative damage • ↓neuro-inflammation	Lum et al., (2023b)
Cognitive Disorder		male Kunming mice		50 mg/kg orally for 11 days	• ↓cognitive deficits • ↓ HO-1 • ↓ IL-6	Fu et al., (2015)
	hippocampal neuronal cell line HT22	old male C57/BL6 mice	25–250 µM	40 mg/kg/day intragastrica-lly for 6 weeks	• ↓cognitive deficits • ↓Tau-associated kinases	Chen et al., (2024)
		Male Sprague–Dawley rats		(15, 30, and 60 mg/kg p.o.) for 8 weeks	• ↑Cognitive performances • ↓oxidative stress	Liu et al., (2013)
		male Kunming mice		12.5 and 50 mg/kg/day for 8 weeks	• ↑PI3K/Akt/Nrf2 signaling • ↓oxidative stress • ↓Apoptosis	Zhang et al., (2023)
		Male young Wistar Rats		20 and 40 mg/kg for 21 days	• ↓memory and motor deficits • ↓oxidative damage • ↓neuro-inflammation • ↓ BDNF depletion	Arora et al., (2020)
Stroke and Ischemic brain injury		Sprague–Dawley rats		(50, 100, 200 mg/kg, intragastrically)	• ↓ Oxidative stress • ↑ PI3K/Akt/mTOR • ↓ Apoptosis	Xi et al., (2018)
	Mouse neuroblastoma (N2a) cells		(100 μM)		• ↑SIRT1/PGC-1α • ↓Oxidative stress • ↓ Apoptosis	Chen et al., (2021)
		C57BL/6 mice		(5, 20 mg/kg, p.o.)	• ↓ NF-κB signalling pathway	Hao et al., (2023)
		Sprague‒Dawley rats		(40 mg/kg, ip)	• Modulation of lipid metabolism	Zhang et al., (2024a)
		Sprague–Dawley rats		(100 mg/Kg)	• ↓ NLRP3-inflammasome	Fan et al., (2017)
Depression	Microglial BV2 cells	Female BALA/c mice	(10,20,40 μM)	(20 and 60 mg/kg, ip)	• ↓ MAPK in microglia	Yan et al., (2022)
		Swiss mice		(40 mg/kg; p.o.)	• ↓ Oxidonitrosative stress • ↓ IDO activity	Luo et al., (2017)
		Swiss mice		(20, 40 mg/kg, p.o.)	• ↓ Oxidative stress • ↓ Neuroinflammation	Jangra et al., (2014)
		Male ICR mice		(20, 40 mg/kg, intragastrically)	• ↓NLRP3-inflammasome • ↓ neuro-inflammation	Cao et al., (2017)
Epilepsy		Swiss albino mice		(25 mg/kg; p.o.)	• Neurotransmitters modulation • ↓ Oxidative stress • ↓ Neuro-inflammation	Li et al., (2024)
Anxiety		Swiss mice		(40 mg/kg; p.o.)	• ↓ Oxidonitrosative stress • ↓ IDO activity	Luo et al., (2017)
		Swiss mice		20 and 40 mg/kg, p.o.)	• ↓ Oxidative stress • ↓ Neuro-inflammation	Jangra et al., (2014)

### Effect of mangiferin on alzheimer’s disease

Mangiferin demonstrates anti-neuroinflammatory effects against LPS-induced inflammation via regulating microglial macrophage polarisation and suppressing the NF-κB and NLRP3 signalling pathways. Moreover, mangiferin treatment significantly reduced the production of NO, IL-1β, IL-6, and TNF-α, in addition to decreasing the mRNA and protein expression of iNOS and COX-22 Mangiferin at a dosage of 40 mg/kg alleviated AlCl3-induced neurotoxicity in the hippocampus by inhibiting oxidative-nitrosative stress, inflammation, acetylcholinesterase activity, and preventing the depletion of brain-derived neurotrophic factor, while also reducing cognitive dysfunction resulting from chronic AlCl3 administration (Kasbe et al. 2015). Mangiferin (40, 20, and 10 mg/kg) significantly improved cognitive learning and memory retention. Pre-treatment with Mangiferin restored raised whole brain acetylcholinesterase levels, lipid peroxidation, and reduced glutathione caused by scopolamine and normal ageing. Mangiferin mitigated the increased levels of dopamine and noradrenaline throughout the brain (Biradar et al. 2012). Mangiferin exhibits neuroprotective effects against A*β* owing to its antioxidant and anti-inflammatory mechanisms. Mangiferin significantly elevates dopamine concentrations in the substantia nigra and alleviates the impact of neurotoxins linked to oxidative stress, mitochondrial dysfunction, and apoptosis (Feng et al. 2019). Furthermore, mangiferin has been shown to improve learning and memory in the Morris Water Maze test, maintain hippocampus cell integrity, reduce the accumulation of brain A*β* species, and decrease levels of brain lipid peroxidation (Du et al. 2019). Mangiferin (20 mg/kg) ameliorated long-term cholinergic memory impairments by acetylcholinesterase inhibition or cholinergic receptor stimulation and suppression of NF-kB activation (Jung et al. 2009). Mangiferin may safeguard against β amyloid-induced neurotoxicity in the Neuro 2A brain cell line, while also inhibiting AChE and 5-LOX, demonstrating considerable antioxidant effects and reversing amnesia (Sethiya and Mishra 2014). The oral treatment of mangiferin significantly reduced tau hyperphosphorylation in the cortex and hippocampus. Moreover, inflammatory processes, evaluated via microglial and astrocytic loads, were reduced in mice treated with mangiferin. Moreover, neuronal morphological alterations, such as aberrant neurite curvature and dystrophies, significantly increased in APP/PS1 mice, were substantially ameliorated by extended mangiferin treatment. The reduction of these pathological traits correlated with notable improvements in episodic and spatial memory in APP/PS1 mice treated with mangiferin (Infante-Garcia et al. 2017).

### Effect of mangiferin on parkinson’s disease

Mangiferin improved motor function and reduced the activation of microglia and astrocytes in a mouse model of Parkinson’s disease. Mangiferin decreased reactive oxygen species (ROS) levels while augmenting glutathione (GSH) and superoxide dismutase (SOD) concentrations. Mangiferin markedly enhanced the expression of GIT1, p-ERK, Nrf2, HO-1, and SOD, while concurrently inhibiting Keap1 expression, both in vitro and in vivo (Zhou et al. 2023). Furthermore, mangiferin exhibits a therapeutic effect against Parkinson’s disease by regulating GIT1 and its downstream Keap1/Nrf2 pathways (Zhou et al. 2023). Mangiferin mitigated MPTP-induced behavioural deficits, oxidative stress, apoptosis, dopaminergic neuronal degeneration, and dopamine depletion. Thus, the overexpression of the anti-apoptotic Bcl-2 protein and the suppression of the pro-apoptotic Bax by mangiferin have been shown to alleviate the neurotoxicity and apoptosis induced by MPTP (Kavitha et al. 2013). Mangiferin decreased α-synuclein accumulation and suppressed AKR1C3 expression, therefore stimulating the Wnt/β-catenin signaling pathway (Huang et al. 2024). Treatment with mangiferin (45 µg/kg) significantly improves key locomotor activity metrics, reduces oxidative stress, and lowers indications of inflammatory stress. The activity of caspases was reduced, and a significant decrease in the activity of both cyclooxygenase 1 and 2 was observed (Tiwari et al. 2021). Researchers found that mangiferin slowed the progression of Parkinson’s disease in zebrafish by maintaining the homeostatic expression of several genes. By controlling the expression of crucial genes such as lrrk2, vps35, atp13a, dnajc6, and uchl1, mangiferin reduces mitochondrial-related oxidative stress in zebrafish models of Parkinson’s disease (Qin et al. 2024). The levels of pro-inflammatory cytokines, COX-2, total nitrite (NOx), and FOS B are reduced in rats with 6-OHDA lesions when treated with 45 μg of mangiferin and 10 mg/kg of 7-NI, either alone or in combination. Locomotor parameters are also dramatically improved (Tiwari et al. 2022). Additionally, mangiferin therapy decreased myeloperoxidase (MPO) levels and alleviated oxidative stress. Accompanied by a decrease in caspase-3, caspase-9, and COX-2 activity in rats with 6-OHDA lesions (Tiwari et al. 2024a). The substantia nigra of MPTP-induced Parkinson’s disease rats may express more tyrosine hydroxylase, and mangiferin may improve motor impairments. In addition, MGF raised mitochondrial ATP levels and improved mitochondrial ultrastructure. Mitochondrial growth factor (MGF) may influence the levels of Drp1 and other mitophagic proteins including PINK1, Parkin, NIX, BNIP3, FUNDC1, and p62 (Wang et al. 2022). By restoring GSH levels, protecting N2A cells from MPP + -induced cytotoxicity, and lowering mRNA expression of SOD1 and CAT, mangiferin proved crucial. Along with ROS neutralization (Amazzal et al. 2007). Pre-treatment with mangiferin markedly improved cell survival, mitigated the reduction in mitochondrial membrane potential, and diminished rotenone-induced apoptosis in a cellular model of Parkinson’s disease (Kavitha et al. 2014). Mangiferin demonstrates neuroprotective properties against H2O2 neurotoxicity through the activation of Nrf2/ARE signaling in PC12 cells (Shi et al. 2017).

### Effect of mangiferin on huntington’s disease

Mangiferin mitigated the histopathological modifications that 3-NP induced in the hippocampus, striatum, and cortex of the brain. Mangiferin’s antioxidant and anti-inflammatory properties appear to protect the brain from oxidative injury and neuroinflammation. Mangiferin demonstrated a significant decrease in brain malondialdehyde (MDA) levels, an increase in reduced glutathione (GSH) levels, and enhanced activities of succinate dehydrogenase (SDH), superoxide dismutase (SOD), and catalase (CAT). Additionally, the levels of tumour necrosis factor-alpha (TNF-α), interleukin-1 beta (IL-1β), and interleukin-6 (IL-6) decreased (Lum et al. 2023b).

#### Effect of mangiferin on stroke and ischemic brain injury

In fact, Xi et al. (2018) have proposed that mangiferin may have neuroprotective properties in a perinatal hypoxic-ischemic brain injury model in rodents. A study has indicated that mangiferin can enhance the neuroprotective effects of isoflurane. It has been reported that the anti-apoptotic proteins Bcl-2 are significantly increased in co-treatment with mangiferin, while the apoptotic proteins Bad and Bax are decreased. Additionally, apoptosis is suppressed. Additionally, the findings of this investigation have demonstrated the function of mangiferin in the neutralisation of ROS, which mitigates oxidative stress by reducing the production of ROS and the level of MDA. In the interim, the administration of isoflurane and mangiferin resulted in an increase in GSH levels. Furthermore, mangiferin enhances the neuroprotective effects of isoflurane by activating the phosphoinositide 3-kinase (PI3K)/protein kinase B (Akt) signalling pathway. The administration of mangiferin has been reported to increase the expression of mTORC2 and *p*-mTORC2, resulting in the phosphorylation and activation of Akt. This process then inactivates GSK-3b and NF-κB, thereby contributing to the amelioration of neonatal ischaemic brain injury.

An earlier study has addressed mangiferin as a potential therapy drug for blast-induced traumatic brain injury (Fan et al. 2017). In rodents, mangiferin exhibited anti-inflammatory and anti-oxidant effects by establishing blast-induced traumatic brain injury models. Mangiferin has been demonstrated to reduce brain damage by reducing ROS/TXNIP and thioredoxin (TRX)-interacting protein. This inhibits the activation of the NOD-like receptor family, pyrin domain-containing 3 (NLRP3) inflammasome, which in turn reduces the activated capaspase-1 and contributes to the reduction of IL-1β. Consequently, oxidative stress is alleviated and endogenous antioxidants are enhanced. Furthermore, mangiferin has been reported to have an anti-inflammatory effect by inhibiting the secretion of TNF-α.

Additionally, Hao et al. (2023) have suggested that mangiferin possesses neuroprotective properties against focal cerebral ischaemia. Mangiferin has been demonstrated to mitigate the brain injury caused by a blocked middle cerebral artery in rodents by administering it at concentrations of 5 and 20 mg/kg. This treatment contributes to the improvement of neurological function, the reduction of brain swelling, and the reduction of infarct size. Mangiferin has been shown to suppress neuroinflammation, thereby attenuating focal cerebral ischaemia. Mangiferin has been demonstrated to significantly impede the nuclear expression of NF-κB p65 and the phosphorylation of IκBα, resulting in the suppression of the activation of the NF-κB signalling pathway. In addition, mangiferin has been demonstrated to reduce the expression levels of a variety of inflammatory mediators, such as TNF-α, IL-1β, iNOS, and COX-2.

A prior study has proven the neuroprotective impacts of mangiferin in ischemic stroke rats (Zhang et al. 2024a). It has been reported that mangiferin modulates lipid metabolism, thereby relieving poststroke cognitive impairment.

An earlier study has suggested neuroprotective effects of mangiferin against cerebral hypoxia/reoxygenation brain injury in neuroblastoma cells (Chen et al. 2021). According to reports, mangiferin prevents oxidative stress, apoptosis, and mitochondrial dysfunction via increasing SIRT1/PGC-1 α signalling, which in turn increases the expression of NRF1, UCP2, and Bcl2. On top of that, mangiferin is known to increase Nrf2 expression, a cytoprotective transcription factor that controls the expression of genes like NQO1 and HO-1 that code for antioxidant enzymes and proteins.

### Effect of mangiferin on epilepsy

A prior study has illustrated the antiepileptic benefits of mangiferin against seizures triggered by the chemical convulsant pentylenetetrazol in mice (Li et al. 2024). Forced swim tests and actophotometer results showed that mangiferin reduced seizure severity and sadness. In addition, seizure-induced mice treated with mangiferin had lower ratings for seizure severity and less convulsions, since mangiferin greatly increased GABA levels and inhibited glutamate synthesis. Also, in the brain tissue of seizure-induced mice, co-treatment with mangiferin increased levels of Na +, K + -ATPase and Ca2 + ATPase. These enzymes are critical for the start and end of convulsions. Mangiferin also showed anti-inflammatory and antioxidant capabilities by lowering levels of nitric oxide and malondialdehyde, increasing the activity of glutathione and superoxide dismutase, and lowering levels of TNF-α, IL-1β, and COX-2, which are cytokines that promote inflammation. Collectively, the neuroprotective effects of mangiferin against epilepsy are thought to be due to its ability to regulate oxidant imbalance, reduce neurotransmitter levels, and inhibit the production of pro-inflammatory cytokines.

### Effect of mangiferin on depression and anxiety

#### Depression

A prior study has revealed the beneficial impact of mangiferin in mitigating postpartum depression in a hormone-simulated pregnancy mice model along with ovariectomy (Yan et al. 2022). Some studies have shown that mangiferin can help reduce symptoms of postpartum depression in mice. Research has shown that mangiferin can reduce inflammation in the brain, which could be a factor in postpartum depression. It has been shown that mangiferin greatly inhibits the MAPK signalling pathway, which is supported by the fact that it lowers the levels of IBAl, a marker for microglia, which in turn reduces microglial activation and the levels of inflammatory cytokines, including TNF-*α*, IL-6, and IL-1ꞵ.

An earlier study has explored the antidepressant effect of mangiferin in corticosterone-treated rats (Luo et al. 2017). Results from the forced swimming test (FST) and the tail suspension test (TST) showed a decrease in immobility time when administered concurrently with mangiferin, suggesting that the corticosterone-mediated depressive-like behaviour was eased. According to reports, mangiferin can improve the neurobehavioral changes caused by corticosterone in four ways: (1) by lowering kynurenine production by suppressing indoleamine 2,3-dioxygenase (IDO) activity in the hippocampus, which in turn reduces the negative effects of corticosterone on the brain; (2) by increasing levels of brain-derived neurotrophic factor (BDNF); (3) by restoring GSH activity and reducing levels of malondialdehyde (MDA); (4) by inhibiting NF-κB activation, neuroinflammation is suppressed, and proinflammatory cytokines like IL-1β and TNF-*α* and nitrite levels are reduced.

The antidepressant action of mangiferin has been hypothesised by Jangra et al. (2014). Research in mice has shown that mangiferin can protect neurones against the changes in behaviour and biochemistry caused by lipopolysaccharide (LPS). Mangiferin reportedly exerts its neuroprotective effects via reducing levels of BDNF in the prefrontal cortex and hippocampus and by blocking chronic inflammation and oxidative stress. It has been demonstrated that mangiferin reduces IL-1β levels, increases BDNF levels, and restores the activity of SOD and catalase. The favourable effects of mangiferin are supported by the fact that it reduces anhedonic behaviour, a hallmark of depression, and by the results of the FST and the TST. A prior study that established chronic mild stress (CMS) in a mouse model indicated that mangiferin possesses antidepressant properties (Cao et al. 2017). Increasing body weight, decreasing sucrose intake, improving locomotor activity, and decreasing immobility time in the FST and TST are all ways in which mangiferin has been shown to display antidepressant effects in CMS mice. Inhibiting the high levels of IL-1β, IL-18, and TNF-*α* in the hippocampus, mangiferin exerts anti-inflammatory and neuroprotective properties, which may help reduce depression symptoms. Meanwhile, it has been shown that mangiferin’s anti-inflammatory effects are mediated by blocking the NLRP3-inflammasome/caspase-1/IL-1β pathway, which is believed to have a role in the development of depression. Additionally, Mangiferin improved hypothalamic–pituitary–adrenal axis dysfunction by lowering CMS mice’s high blood corticosterone levels.

An in vitro study suggested the inhibitory role of mangiferin on microglia-mediated inflammation, participating in the amelioration of postpartum depression (Yan et al. 2022). Mangiferin has been shown to reverse the LPS-induced boost in iNOS, *p*-JNK, and *p*-p38 levels, thereby blocking the MAPK signalling pathway and reliably reducing the levels of the downstream inflammatory cytokines TNF-*α*, IL-6, and IL-1ꞵ.

#### Anxiety

An earlier study demonstrated the neuroprotective effect of mangiferin in a corticosterone-induced anxiety model in mice (Luo et al. 2017). Results from the elevated plus maze (EPM) and the light–dark exploration tests show that mangiferin reduces anxiety-induced behavioural abnormalities. Reducing the kynurenine/tryptophan ratio confirms that mangiferin reduces anxiety via raising BDNF levels and lowering IDO-induced kynurenine levels in the hippocampus. Research has shown that mangiferin can help reduce inflammation in the hippocampus and oxidative-nitrosative stress caused by corticosterone. It also has anti-inflammatory and antioxidant effects. Reasons given for mangiferin’s positive effects include lowering MDA levels, restoring GSH activity, inhibiting NF-κB activation, and lowering nitrite and proinflammatory cytokine levels (IL-1β and TNF-*α*).

An earlier study has suggested the neuroprotective effects of mangiferin on lipopolysaccharide-induced anxiety-like effects in mice (Jangra et al. 2014). Evidence from the open field test, elevated plus maze, and light–dark box studies shows that mangiferin has anti-anxiety benefits. Inhibition of neuroinflammation, oxidative stress, and maintenance of BDNF levels in the brain of mice are the mechanisms linked to mangiferin’s neuroprotective benefits. According to reports, mangiferin reduced IL-1β levels, increased BDNF in the hippocampus, decreased nitrite levels, and reduced the anti-oxidant activity of enzymes such as SOD and catalase.

### Effect of mangiferin on cognitive disorders

#### Memory and learning enhancement

Mangiferin may demonstrate neuroprotective properties against LPS-induced cognitive impairments. Moreover, mangiferin may modulate HO-1 expression, thus inhibiting the release of pro-inflammatory factors and reducing LPS-induced IL-6 production (Fu et al. 2015). Murine hippocampal cells (HT22) co-treated with MGF significantly attenuated FA-induced cytotoxicity and dose-dependently inhibited Tau hyperphosphorylation. The protective effects were also demonstrated to be achieved via reducing FA-induced endoplasmic reticulum stress (ERS), as evidenced by the downregulation of ERS markers GRP78 and CHOP and the upregulation of downstream Tau-associated kinases GSK-3β and CaMKII. Moreover, MGF markedly reduced FA-induced oxidative damage, which includes ERS-related Ca2 + excess, ROS generation, and mitochondrial dysfunction. Afterwards, studies showed that C57/BL6 mice with FA-induced cognitive deficits significantly improved their spatial learning and long-term memory after receiving 40 mg/kg/day of MGF intragastrically for 6 weeks. This improvement was brought about by a decrease in Tau hyperphosphorylation as well as levels of GRP78, GSK-3β, and CaMKII in the brain tissue (Chen et al. 2024). Reductions in escape latency and increases in platform crossings and target quadrant percentage show that mangiferin significantly improved the behavioural performance of diabetic rats. In diabetic rats, this was linked to lower levels of advanced glycation end-products and their receptor (RAGE), interleukin-1β, tumour necrosis factor-α, and malondialdehyde, as well as higher levels of glutathione and enhanced activity and expression of glyoxalase 1 (Liu et al. 2013). Mangiferin may also interact with proteins linked to age-related oxidative stress and cell death. In an experiment with aged rats, mangiferin successfully reduced the histological changes brought on by D-gal in the brain and liver. In addition to lowering AChE and MDA levels in blood, liver, and brain tissues, mangiferin treatment greatly increased the activity of antioxidant enzymes (SOD, T-AOC, GSH-Px, and CAT). In addition to improving exploratory abilities, MAN reduced learning and memory deficits. In hepatic tissues, mangiferin may also regulate the expression of apoptotic proteins (Bax, Bcl-2, and Caspase-9) and proteins linked to the PI3K/Akt/Nrf2 signalling pathway (Zhang et al. 2023). Animals given mangiferin treatment showed significant improvement in motor and cognitive deficits compared to animals given QA. Moreover, the levels of MDA, nitrite, and pro-inflammatory cytokines were lowered in the striatum and hippocampus, and the GSH concentration was restored following treatment of mangiferin (40 mg/kg). Acute cholinergic dysfunction (AChE), loss of brain-derived neurotrophic factor (BDNF), and mitochondrial dysfunction were all ameliorated by MGF treatment (Arora et al. 2020).

## Animal and human toxicity

Mangiferin has demonstrated low toxicity profiles in both in vitro and in vivo studies. In numerous rodent models, doses ranging from 10 to 200 mg/kg administered orally or intraperitoneally over periods of 14–60 days have not shown any signs of acute or subchronic toxicity, including neurotoxicity. Instead, mangiferin consistently exerted beneficial effects on cognitive function, locomotor activity, and behavioral parameters, indicating favorable CNS tolerability (Kavitha et al. 2013; Du et al. 2019; Lum et al. 2023b).

In vitro studies using neuronal cell lines such as PC12, N2A, SH-SY5Y, and HT22 confirm that mangiferin does not induce cytotoxicity at concentrations up to 100 μM. On the contrary, it protects against various neurotoxins (e.g., MPP +, formaldehyde, and rotenone) by reducing apoptosis, mitochondrial dysfunction, oxidative stress, and inflammatory signaling (Chen et al. 2024; Shi et al. 2017; Kavitha et al. 2014). These findings support its CNS safety and therapeutic potential.

With respect to human application, mangiferin is a naturally occurring xanthone C-glucoside primarily found in *Mangifera indica* (mango) and is commonly consumed as part of the human diet. While mangiferin itself is not yet approved as a standalone pharmaceutical by major regulatory authorities such as the US. FDA or EMA, it has been included as a bioactive ingredient in several over-the-counter nutraceutical formulations due to its antioxidant, anti-inflammatory, and antidiabetic properties. It’s generally recognized safety in food sources and as a nutraceutical further supports its translational potential.

However, clinical data are currently limited, and further studies including dose-escalation and long-term safety evaluations in humans are warranted. Future clinical trials should assess pharmacokinetics, blood–brain barrier permeability, and potential drug interactions to fully characterize its safety and efficacy profile in human neurological conditions.

## Pharmacokinetics and bioavailability and potential therapeutic applications

Mangiferin, a naturally occurring phytochemical compound (polyphenol) found in the mango tree (Mangifera indica), has demonstrated substantial neuroprotective properties. Studies have reported its effectiveness in protecting neuronal cells from damage induced by oxidative stress and inflammation, both of which are prominent in promoting neurological disorders, including Alzheimer’s and Parkinson’s (Zivković et al. 2024). Mangiferin possesses various advantages over the current drugs on the market. Initially, it has a diverse pharmacological profile, which includes antioxidant, anti-inflammatory, and anti-apoptotic activities that contribute to its neuroprotective amenities (Shivam and Gupta, 2024). Mangiferin has been demonstrated to affect many molecular targets, such as NF-κB, Nrf2-HO-1, and PI3K/Akt pathways, which are critical in the etiology of neurodegenerative disorders (Turkar et al. 2024). In contrast, many market drugs used for treating neurological conditions target an individual molecular pathway and may render serious side effects such as GIT and cardiac problems, liver and kidney damage, cognitive impairment and mood swings (Xiang et al. 2024).

Nevertheless, there are limitations with its absorption and metabolism in the central nervous system (CNS). Mangiferin is extensively metabolized in the liver and gastrointestinal tract following ingestion, which drastically lowers its bioavailability (Iqbal et al. 2024). This indicates that a small fraction of mangiferin that is consumed reaches the bloodstream and then the CNS, revealing its low concentration in the CNS (Mahmood et al. 2025). Several enzymes are involved in the metabolism of mangiferin, further breaking it down into smaller metabolites, which may or may not retain the inherent biological activities of mangiferin. These metabolites may be further broken down in the liver or excreted through urine (Fuentes-Rios et al. 2025).

Enhancing mangiferin’s bioavailability in the CNS is crucial for maximizing its therapeutic potential. Multiple approaches have been proposed to address the challenges related to its absorption and administration (Samadarsi et al. 2024). One promising method is the establishment of mangiferin derivatives. Mangiferin’s chemical structure is being modified in order to increase its solubility, stability, and overall pharmacokinetic profile (Shah et al. 2025). These compounds may improve absorption and distribution throughout the body, elevating the amount of mangiferin that reaches CNS.

Mangiferin derivatives have been studied recently and compared to the parent molecule, 3,6,7-tribenzoyl-mangiferin, typically of which the latter showed similar to better analgesic and anti-inflammatory activities than the parent mangiferin (Ahmad et al. 2024). Similarly, mangiferin -2′,3,3′,4′,6,6′,7-heptasulfate was found to be a more potent anticoagulant than the parent molecule (Ehianeta et al. 2016).

Many other modifications were conducted aiming to increase mangiferin bioavailability, such as glycosylating the mangiferin using Arlhrobacler nicolianae (da Rocha Ferreira et al. 2013), sugar esters, lipase derivatization (He et al. 2015).

Many studies are adopting such techniques to increase the therapeutic outcomes of mangiferin. This was efficiently achieved by complexation with a phospholipid (Lipoid E80) at 1:1 molar ration; such modification produced a complex with completely different and fortunately higher mangiferin permeability (Khurana et al. 2017).

Also, co-administration of mangiferin with permeation enhancers is another option being researched. These enhancers can temporarily breach the blood–brain barrier (BBB), rendering mangiferin more easily absorbed into the CNS. This approach must be carefully monitored to prevent damaging the BBB and ensuring the safe distribution of mangiferin (Li et al. 2008). The exact mechanism by which mangiferin cross the BBB is still unknown; however, some studies suggested that the daily oral administration of Mangifera indica L. extract crossed the BBB and offered neuronal protection (Liu et al. 2012). Magniferin levels following a single oral dose of Rhizoma Anemarrhenae extract were investigated, in which traces of the drug were detected in the brain (Long et al. 2020).

Meanwhile, the generation of prodrugs is another approach for increasing mangiferin’s bioavailability. Prodrugs are inert chemicals that transform into active drugs once inside the body (Lum et al. 2023a). Prodrugs can boost mangiferin distribution to the CNS by avoiding specific metabolic pathways, hence increasing its therapeutic efficacy. Mangiferin was believed to function as a prodrug, being converted into a number of different metabolites at a particular location in the body that may have more potent biologic effects than the original substance (Ma et al. 2014). Mangiferin was found to be de-glycosylated by intestinal bacteria to Norathyriol, with lower antioxidant but much higher anticancer activity against colon cancer than the parent molecule. Norathyriol, being an expensive molecule, no studies have been performed yet to compare its oral bioavailability to Mangiferin, although research points towards its possible higher bioavailability (Sánchez et al. 2001).

Furthermore, intranasal drugs’ administration is a non-invasive approach that has attracted attention due to its ability to deliver drugs directly into the brain. Mangiferin, when administered intranasally, can circumvent the BBB and reach the CNS more efficiently (Souza et al. 2020). This approach is a promising alternative to established oral and intravenous means of administration. Intranasal application of polysorbate-80-coated Mangiferin PLGA nanoparticles was used to treat cerebral ischemia (de Souza et al. 2013). This formulation efficiently achieved a high drug level in the brain while escaping first-pass effect. Large drug payload (76.08 ± 4.91%) with controlled and sustained release of mangiferin with prolonged retention time and high permeation (> 83%) directly to the brain treating cerebral ischemia at very small drug doses, are fruitful outcomes achieved by this study.

Moreover, mangiferin’s therapeutic efficacy can potentially be augmented through combination therapy, in which it is coupled with other compounds having neuroprotective properties with synergistic properties (De Souza, 2012). This method may assist overcome some of the bioavailability difficulties associated with mangiferin and increase its overall efficacy in treating CNS-related conditions. These tactics represent continuing research efforts to maximize mangiferin’s therapeutic benefits.

A combination of mangiferin with nitro-*L*-arginine methyl ester (*L*-NAME) achieved synergistic neuroprotective activity, improved the forelimb akinesia and motor function in experimental rats. Such a combination statistically decreased the oxidative stress level that exacerbates the neurological damage in animals. Similar results were achieved when combining levodopa with mangiferin (Tiwari et al. 2024b). The anticancer effect of mangiferin was found to be significantly potentiated when combined with gallic acid (Wang et al. 2010).

Additionally, advanced drug delivery technologies, such as nanoparticles, liposomes, and micelles, have also shown promise in increasing mangiferin’s bioavailability (Yang et al. 2013). These systems can encapsulate mangiferin, preventing it from degradation and increasing its absorption. Furthermore, they can be tailored to target specific tissues, such as the brain, boosting the concentration of mangiferin in the CNS.

Citric pectin, pumpkin pectin and chitosan are examples of natural polymers used to encapsulate mangiferin using the spray drying technique (Siddiqui et al. 2024). The nature of polysaccharide, its charge and the type of surfactant used greatly impact the drug retention in the particles; where positively charged chitosan was found to have three times lower drug retention capacity for mangiferin than the negatively charged pectins.

Inclusion of mangiferin in B-cylcodextrin was found to be useful in increasing the bioavailability, thermal stability and water solubility of the drug payload (Alshati et al. 2023).

A phospholipidic complex of mangiferin was synthesized and subsequently incorporated into NLCs made of Compritol 888 as the solid lipid and Labrafil M 2125 as the liquid lipid. The system showed a high encapsulation efficiency and offered a sustained release lasting up to 10 h. Additionally, compared to mangiferin alone, these NLCs more effectively inhibited the efflux activity of *P*-gp in Caco-2 cells, leading to a significant increase in all: the area under the curve, time to peak absorption (tmax), and peak plasma concentration (Cmax) (Kumari et al. 2021).

Mangiferin was also formulated in NLCs based on Labrasol, Glyceryl monostearate, Tween 80 and Miglyol (Santonocito et al. 2023). Such a combination succeeded in increasing the ophthalmic bioavailability of mangiferin 5.69-fold compared to the drug solution.

By investigating the challenges of mangiferin’s bioavailability and CNS delivery, current research hopes to unlock the full potential of mangiferin in combating several neurodegenerative disorders as a promising phytochemical compound as well as other CNS disorders.

## Conclusion

Mangiferin is a potential phytochemical with outstanding neuroprotective potential in various neurological disorders. Through its mechanisms of action involving antioxidant activity, anti-inflammatory effects, protection against mitochondrial injury, and modulation of neurotransmitters, mangiferin successfully opposes pathophysiologic processes involved in neurodegeneration and neuroinflammation. Clinical usefulness is, nonetheless, compromised by limited permeability across the CNS along with poor bioavailability.

To counter these issues, advanced methods such as chemical modifications, nanoparticle encapsulation, prodrug formation, and intranasal delivery have been studied and shown encouraging outcomes in terms of enhancing the pharmacokinetic profile of mangiferin. Moreover, combination therapies and synergistic formulation enhance its applicability as a versatile neuroprotective compound.

Future research should target taking preclinical information to the level of clinical trials, optimizing delivery methods, and establishing long-term safety and efficacy profiles. With further research, mangiferin may provide a novel, effective, and tolerable treatment for managing numerous neurodegenerative and neuropsychiatric conditions.

a polyphenolic xanthone naturally present predominantly in Mangifera indica (mango). The compound has been of interest to scientists due to its remarkable antioxidant, anti-inflammatory, and anti-apoptotic activity. Such bioactivities make mangiferin a promising therapeutic agent for many neurodegenerative and neuropsychiatric disorders such as Alzheimer’s disease, Parkinson’s disease, Huntington’s disease, ischemic stroke, epilepsy, anxiety, depression, and overall cognitive impairment.

The neuroprotection offered by mangiferin is the result of its multi-targeted modes of action. It modulates key inflammatory and oxidative stress pathways such as NF-κB, NLRP3, PI3K/Akt, and Nrf2/ARE. It also influences signaling pathways involved in neuroplasticity and neurotransmission, such as the MAPK cascade and BDNF regulation. These mechanisms allow mangiferin to intervene at several pathological checkpoints in neuronal injury and disease development.

The compound’s pharmacological benefits are supported by in vitro and in vivo studies, demonstrating that mangiferin attenuates neuroinflammation, suppresses oxidative stress, prevents tau hyperphosphorylation, limits amyloid-beta deposition, and protects mitochondrial integrity. It also improves motor coordination, cognition, and neurotransmitter balance in animal models of neurodegeneration. In addition, mangiferin structure–activity relationship (SAR) studies reveal that C-glucosylation and the presence of hydroxyl groups are essential for its efficacy.

Despite its therapeutic promise, mangiferin’s clinical application is hindered by its poor bioavailability and low ability to cross the blood–brain barrier. To bypass these limitations, researchers are exploring various drug delivery methods such as nanoparticle encapsulation, prodrug synthesis, phospholipid complexes, and intranasal administration. These are aimed at enhancing its stability, absorption, and CNS targeting.

Overall, mangiferin is a potent, multi-target neuroprotective drug with the potential to intervene in multiple pathways of neurodegenerative diseases. While preclinical data are encouraging, more studies—particularly clinical trials and improved drug delivery systems are needed to achieve its successful therapeutic use in humans.

## Data Availability

Not applicable.
